# “They make it too hard and too many hoops to jump”: system and organizational barriers to drug treatment during epidemic rates of opioid overdose

**DOI:** 10.1186/s12954-024-00964-5

**Published:** 2024-02-27

**Authors:** Julia Dickson-Gomez, Sarah Krechel, Jessica Ohlrich, Helena Danielle Green Montaque, Margaret Weeks, Jianghong Li, Jennifer Havens, Antoinette Spector

**Affiliations:** Medical College of Wisconsin, Milwaukee, USA

**Keywords:** Medications to treat opioid use disorder (MOUD), Opioid use disorder, Heroin, Fentanyl, Overdose, Detox, Medically assisted withdrawal, Polysubstance use

## Abstract

**Introduction:**

The United States is currently facing an opioid overdose crisis. Research suggests that multiple interventions are needed to reduce overdose deaths including increasing access and retention to medications to treat opioid use disorders (MOUD, i.e., methadone, buprenorphine, and naltrexone) and increasing the distribution and use of naloxone, a medication that can reverse the respiratory depression that occurs during opioid overdoses. However, barriers to MOUD initiation and retention persist and discontinuations of MOUD carry a heightened risk of overdose. Many times, MOUD is not sought as a first line of treatment by people with opioid use disorder (OUD), many of whom seek treatment from medically managed withdrawal (detox) programs. Among those who do initiate MOUD, retention is generally low. The present study examines the treatment experiences of people who use opioids in three states, Connecticut, Kentucky, and Wisconsin.

**Methods:**

We conducted in-depth interviews with people who use opioids in a rural, urban, and suburban area of three states: Connecticut, Kentucky and Wisconsin. Data analysis was collaborative and key themes were identified through multiple readings, coding of transcripts and discussion with all research team members.

**Results:**

Results reveal a number of systemic issues that reduce the likelihood that people initiate and are retained on MOUD including the ubiquity of detox as a first step in drug treatment, abstinence requirements and requiring patients to attend group treatment. MOUD-related stigma was a significant factor in the kinds of treatment participants chose and their experiences in treatment.

**Conclusions:**

Interventions to reduce MOUD stigma are needed to encourage MOUD as a first course of treatment. Eliminating abstinence-based rules for MOUD treatment may improve treatment retention and decrease overdose risk.

## Background

The first wave of the current US overdose epidemic involved increased prescription opioid mortality in the 1990s, followed by a second wave of increases in deaths involving heroin around 2010 [1–3]. The current third wave is characterized by illicitly manufactured fentanyl driving overdose mortality rates to record highs. Almost half (47%) of persons who inject drugs (PWID) are estimated to have one or more nonfatal lifetime overdoses [4] and 50–58% of PWID report witnessing a prior year fatal or non-fatal overdose of a peer [4]. Currently in most parts of the United States, fentanyl is most common in the white powder heroin market [5]. Fentanyl is more potent than heroin, with more rapid onset of action and higher overdose risk; fentanyl has a shorter half-life, and overdose, when it occurs, may be immediate, with a shorter window for intervention than a heroin overdose [6]. Fentanyl is now also appearing in methamphetamine, cocaine, and benzodiazepines; polysubstance overdoses have increased greatly over the past few years in part due to the addition of fentanyl to these street drugs and counterfeit pills [7, 8]. Fentanyl and other synthetic opioids now account for the majority of US overdose deaths [5].

Research suggests that multiple interventions are needed to reduce overdose deaths including increasing access and retention to medications to treat opioid use disorders (MOUD, i.e., methadone and buprenorphine) and increasing the distribution and use of naloxone, a medication that can reverse the respiratory depression in opioid overdoses [9]. MOUD is the gold standard for treating OUD [10]; however, fewer than 20% of people with OUD receive MOUD, including methadone or buprenorphine [10–15]. A number of barriers to accessing MOUD have been found in the literature including: stigma against MOUD by both people with OUD and drug treatment providers [16–18], and insurance and regulatory burdens imposed on MOUD [19, 20]. Methadone can only be provided in highly regulated Opioid Treatment Centers to which most patients must travel to receive their daily doses. Prescribing buprenorphine until recently required providers to apply for a waiver to prescribe on an outpatient basis, included specific training, and allowed providers to treat a limited number of patients at any given time [20, 21]. Efforts to increase the number of buprenorphine waivered providers have had some success [22–24]. However, around 71% of counties in the US do not have publicly available MOUD prescribers and almost 60% do not have buprenorphine-waivered providers [25, 26]. Regulations to prescribe buprenorphine and methadone were relaxed during COVID-19 [27, 28]. In 2021 training requirements were removed although providers were still required to apply for a waiver to prescribe. In 2022, the requirement to apply for a waiver was removed altogether.

Less research has focused on retention in MOUD. Retention in MOUD is generally low, although highly variable, from 19%-94% at 3-month, 46–92% at 4-month, 3–88% in 6-month, and 37%-91% at 12-month follow-up in randomized controlled trials [29]. While there are currently no recommended treatment durations for MOUD, longer duration of treatment is associated with better treatment outcomes and lower risk of opioid overdose [30]. Research suggests many program-related policies that may lead to discontinuation of MOUD, including discharge from clinics for missing an appointment or using other substances [31]. Some MOUD clinics require abstinence from all substances and patients who fail to achieve this are discharged from care [32]. Receiving higher doses of MOUD was also associated with better treatment retention, suggesting that some patients may not receive an adequate dose to control cravings [32]. In response to these poor rates of retention, some studies have implemented low-barrier MOUD treatment with softened rules focusing more on retention than abstinence; these have shown similar rates of retention as conventional MOUD treatment while engaging a higher risk population [33, 34]. Future research to determine whether removal of such barriers improves longer-term retention are needed.

Many patients, however, do not seek MOUD as a first course of treatment, but rather seek treatment through inpatient medically managed withdrawal programs. More than 400,000 people with OUD were admitted to detox in 2015 [35, 36]. Detox without further treatment has not been found effective in reducing opioid use or overdose, and many studies have found a considerable risk of overdose for those leaving detox [37, 38]. Further, multiple studies have shown poor linkage to follow-up care, with fewer than half of patients successfully transitioning to any kind of drug treatment [39–45]. Those who do not receive follow-up care are more likely to be quickly readmitted to detox [42, 45–47] while those who do transition to other treatments often face gaps in time between leaving detox and entering drug treatment, with increases in overdose deaths during those transition times [47]. Finally, it is common for patients to leave detox against medical advice [48]. There is considerable disparity between those who enter detox as a first treatment for OUD and those who receive follow-up care, with Hispanics, Blacks, those who are experiencing homelessness or who are on Medicaid more likely to enter detox, to have no follow-up care and to leave against medical advice [6, 7, 43]. While there have been some calls to integrate MOUD into detox with immediate linkage to community-based MOUD [35], it is not entirely clear how easily this could be done [49] and few detox centers currently initiate MOUD in their programs [35, 50, 51]*.*

The present study examines the treatment experiences of people who use opioids in three states, Connecticut, Kentucky, and Wisconsin. Results reveal a number of systemic issues that reduce the likelihood that people initiate and are retained on MOUD including the ubiquity of detox as a first step in drug treatment, abstinence requirements and requiring patients to attend group treatment.

## Methods

### Study overview

The current study is part of a larger project that aims to compare the factors that influence the effects of opioid-related laws and policies in Connecticut, Kentucky and Wisconsin on the transitions from prescription opioids (POs) to heroin, fentanyl, and/or injection drug use. The current paper draws from interviews with participants from urban, suburban and rural areas of each state who use heroin, illicit fentanyl or PO nonmedically. Initial participants were recruited from harm reduction services or upon entry to drug treatment facilities that were identified in key informant interviews. Subsequent participants were referred to the study by people who use opioids who were interviewed through snowball sampling. Eligibility criteria included being 18 years or older and using prescription opioids nonmedically or using fentanyl or heroin in the past 6 months. Participants were compensated $35 for completing in-depth interviews. We conducted 60 in-depth interviews with people who use opioids in Connecticut, 32 in Kentucky and 56 in Wisconsin.

Interviews with people who use opioids were conducted by research staff and students (10 total, three in each state, three students) trained in qualitative interviewing between December 2019 and August 2021. Interviewers were ethnically and racially diverse with three identifying as Hispanic and Spanish speaking and two African American. Interviews focused on their experiences with drug treatment. Participants were asked about the kinds of drug treatment they have experienced, including MOUD, and whether they felt they received the help they needed in them. Participants were probed for relapses and their triggers, and discharge without completing drug treatment. Participants were also asked about any experiences they had with MOUD bought on the street. All study procedures were approved by the Institutional Review Board (IRB) (PRO00030281) of the Medical College of Wisconsin which served as IRB for all sites. All participants provided informed consent before participating in an interview.

### Data analysis

All interviews were transcribed verbatim. We used a collaborative approach for data analysis. To develop a coding tree, we selected a transcript which the multi-state research team read to develop a preliminary list of codes. The preliminary coding list was then applied to three additional transcripts—which were purposively selected to reflect different experiences (e.g., state in which the participant lived, rural or urban location)— and refined until the research team reached consensus on a final list of codes, their meanings, and the procedures for assigning them to text data. The research team then used MAXQDA software to apply the final list of codes to the transcripts. The coding was completed by six members of the multi-state research team. Coding, the development of new codes, and memoing (jottings done by coders to capture relationships between codes or initial hypotheses) were tracked by the six-person team. We used bi-weekly team meetings for troubleshooting and quality checks that included the principal investigator of the study. We also read each transcript to summarize the person’s drug use trajectory, including any experiences with drug treatment. These transitions were examined and compared across participants to discover patterns.

## Results

When asked about their experiences with drug treatment, 24 participants mentioned entering detox centers for medically managed withdrawal from heroin, many more than once. Many considered detox as a first step to continuing drug treatment, including longer-term residential treatment, intensive out-patient or MOUD. In fact, a detox center in the Milwaukee area was called “First Step.”

Interviewer: And now currently, do you think it’s easy to get drug treatment programs in the area?

Participant: Yeah. You just have to be willing to go to detox. You have to be willing, when you’re at detox to say, hey, I want further treatment and they’re pretty helpful on getting you into a program… I mean you got to call and find out what detox has a bed available. Once you’re in the door at detox though, you get assigned a counselor or a caseworker or whatever and they do everything. If you’re just there to lay in bed and detox yourself for five to seven days and go back out and keep using, then that’s what’s gonna happen. But if you really want help, they’ll get you into a 30-day program or a 90-day program. Then after that, they’ll put you in housing. They’ll get your Food Stamps back on, get your IDs, your Social Security, birth certificate back if you don’t have any of it. It’s just you got to take the help if you want it. (HAR201)

While some people mentioned that detoxes were helpful in getting them connected with follow-up care, including residential and MOUD, others reported being released after 5 days.

Participant: I think they’re helpful, but I don’t like the detoxes. I think the detoxes need to do something different because if you go in for a detox, a full detox off of opiates is gonna be at least…30 days before your body is feeling completely back to normal before you even picked it up. The detoxes, they have you going right off the streets from using every day, and they keep you there five to seven days. You’re not even done detoxing by the time they boot you out the door.

Interviewer: So, do they give you a plan or something to continue on the treatment?

Participant: …. No, that’s just it. They’ll give you Suboxone [buprenorphine with naloxone] for the five days that you’re there or methadone or whatever you prefer to use from detox, but they kick you out the door and you go home. And your first night sleeping home, you’re back to the pain and everything’s back, so what are you gonna do? You’re gonna run back out and use. (HAR203)

Many participants considered detox to be drug treatment, although recognizing that it did not seem to work to get them into recovery. They felt that having more time in detox facilities would give them a better chance at abstinence and recovery.

Detox programs may be appropriate for those who want to try abstinence-based programs.

Participant: First, I went to the detox. Then I went to the rehab. They were giving me Suboxone for 5 days. The fifth, I just stopped completely. Then when I went to the rehab they like they want me to put you in treatment. I'm like no. I just wanta s-, I don’t want no Suboxone 'cause even though you can get withdrawals without the Suboxone. So, I'm like zero. The doctor started looking at me and started laughing, so they just drug test me every week to see if I was doing something else and when the – all the, the urine came up clean. They were like wow. I'm like yeah. I don’t need no treatment. I'm [gonna] do it to do a change. (TOL201)

Much of the reluctance to MOUD may come from the stigma that many people have toward it, including providers and people who use drugs. Many participants felt that MOUD was just another drug, feared side-effects of MOUD or worried about withdrawal from methadone and buprenorphine as this participant reported.

Participant: Methadone I know a lot of people that do well on it, but they say the withdrawals are horrible from it. I’ve been considering maybe looking into it. I don't know really the difference in what makes one right for one person and one right for another person. Everyone always told me like methadone is just like legal heroin and you know they have a harsher stigma on it – although people have a pretty harsh stigma on Suboxone too and I don’t get it because it really does help, but Suboxone as well I hear that the withdrawal is worse than dope. You might as well just you know be doing heroin, blah, blah, blah. It’s all the stuff that’s told to you. So, I just don’t really know. They don’t really talk about. You don’t get to go in somewhere like when you go in for birth control and they’re like, “This is an IUD. This is a birth control pill. Which one’s gonna work right for you?” You know you just kind of have to try it and fail at them and see what works. (WAU 214)

However, it is not clear whether participants always went to detox with the idea of continuing in abstinence-based programs or to start naltrexone (a non-opioid MOUD). In fact, many were connected with MOUD directly from the detox center and starting MOUD seemed to be participants’ intention upon entering detox. When asked whether it was difficult to get into a methadone program, the participant below reported:

Female: No, not at all. I went right – got right into a detox the day I called. I didn’t have to wait for the five days and then I did my five-day detox and then I went right to the clinic. They set me up for when I got out. (TOL203)

Entering detox in these situations may help providers buy time to link patients with MOUD programs and to get them out of crisis situations. However, detox is not technically needed if patients are starting methadone or buprenorphine. Patients must be in moderate withdrawal to start buprenorphine to avoid precipitated withdrawal, but complete withdrawal is not necessary. Ironically, in some cases in which detox may have been medically necessary, e.g., polysubstance use with alcohol or benzodiazepines, participants reported not being admitted to detox.

I tried to go to [Name of Clinic] to detox there, and because I had the Xanax problem with the heroin, they were like, “Yeah, we’re not helping you.” When the one detox place that everybody goes to tells you they can’t handle you, that’s not a good sign (MKE203)

In addition to many detox centers not linking patients to continuing treatment, many participants reported stigmatizing and traumatic experiences at detox that led them to leave against medical advice even before their 5 days were completed.

Participant: I didn’t even finish the detox. It was supposed to be seven days, and I lasted one night. I went home the next morning and I tried to continue the detox at home by myself.

Interviewer: Do you think you got the help that you needed at the time?

Participant: No. That’s why I left the next morning. Because they, at the detox where I was at, that whole day I was detoxing and that night, literally throwing myself on the floor because my legs were so bad. They just sat there and watched me. And one of the staff like belittled me, “You put yourself in this position, now suck it up.” Yeah, they weren’t very comforting at all… That’s why I said I’ll try it myself. I came home. I tried detoxing. I tried continuing to detox for two more days…at my house. I couldn’t, so I relapsed. I went out and bought a couple of bags of fentanyl and then I went to the clinic. I remember it because… I came home on a Friday, so Monday morning I went to the clinic and I tried getting on methadone. It took me a few days (HAR204).

While this participant entered methadone shortly after relapsing, negative experiences in drug treatment can cause some to give up on drug treatment all together.

Participant: I was in, I’m not gonna lie, I was in detox three months ago…. I got there 9:00 in the morning. By 11:00, I was admitted in a room, everything. They told me that they were gonna give me the methadone, but I had to wait an hour, right? So, I went in my room and I fell asleep. Do you know, they did not wake me up for meds? They didn’t wake me up for lunch. They didn’t wake me up for supper. They let me sleep throughout the whole night. I got up at 1:00 in the morning, throwing up, diarrhea right in front of the nurses and everything and I said, “Can you guys please medicate me now? You guys did not medicate me all night. You didn’t wake me up. You didn’t even give me my psych meds.” And, “No, we can’t medicate you. You have to wait until meds.” So, they gave me comfort meds. So, I went back to sleep. After three hours of tossing and turning, I got back up at 7:00 in the morning. I’m crawling to the nurse. I couldn’t move. I’m throwing up everywhere. I’m shitting everywhere…. Deathly sick, like whiter than a piece of paper. And I said, “Can you guys please medicate me?” I said, “It’s going on 24 hours that you haven’t medicated me.” And they, the lady turned around and said, “No, you have to wait until 9:00 when it’s med time.” I said, “No.” And now, I’m going crazy. “You’re gonna medicate me now or you’re gonna sign my discharge papers.” And the nurse turned around to me and she goes, “Well, I guess I’m signing your discharge papers, bitch.” The nurse called me a bitch.

Interviewer:

So, so why did you decide to stop – you went to detox. Any reason why or…

Participant: I just wanted to clean my life up. But after that experience, I just don’t, like I don’t even really care (HAR205).

Other participants complained that some county-run detox centers, for people without insurance, were of very poor quality and also treated people with serious mental illness.

Participant: You need to have insurance. Otherwise, it’s really hard. There aren’t a lot of detox places that you wanna go to because the one that’s for free without insurance is just a shithole. It’s actually disgusting…. You’ve gotta wear a hospital gown. The one time I had a hospital gown, it had stains all over it. You walk around with no shoes and stuff like that. There are mental health patients there too. You’re trying to get better on one thing, and you’ve got some other guy that’s yelling or screaming or whatever the case may be. The one time, I was in bed sleeping and I woke up, and a guy was standing above me. You don’t wanna deal with that when you’re dope sick. I’ve also been to some pretty good ones when I had state insurance (MKE205)

### MOUD rules and regulations

Approximately 11 participants had started methadone or buprenorphine but were discontinued from treatment. Many programs require abstinence from other drugs, and participants reported being kicked off MOUD because their urine screens were “dirty”, i.e., showed that they had been using opioids or other drugs. In some cases, participants took drugs which might interact with MOUD and decrease their safety, like benzodiazepines. While this is understandable if patients are using benzodiazepines recreationally, some were prescribed their medication for anxiety like the patient below.

Female: Well, I'm on SSI because I'm – I have bipolar 1, PTSD, anxiety disorder, and so-, social anxiety…. Even though my doctor, my doctor – even though I done drugs in the past and for a long time on and off, I just got off the methadone about a month ago. I went from 80 to 18 and then as soon as they found I was on Klonopin three times a day, they threw me off. My doctor gave them to me because I have such bad anxiety, and I can't control it so... (TOL208)

Some MOUD providers, however, would not allow use of any drugs, including marihuana which does not have dangerous interactions with MOUD. In some cases, being discontinued from MOUD led participants to buy methadone or buprenorphine on the street to control their drug use.

Interviewer: Right. What’s some reasons that people you know are buying suboxone or methadone off the street?

Participant: I don’t know… I guess if anyone smokes pot and stuff, they can’t get into the clinic because of the pot. That’s the reason because they won’t quit…. You can’t take nothing – pot or nothing – when you take those. You can’t take nothing. You get some people out here on meth, and if you go into a clinic on meth, they’ll tell you to go back out the door. Suboxone won’t help you get off meth at all, so they just tell you go on back out the door. (HAZ203)

Most participants, in fact, had bought buprenorphine off the street. However, this is not generally sustainable without a prescription. Many tried to use buprenorphine to manage withdrawal themselves to become, eventually, abstinent from drugs.

Participant: They’ve [friends and acquaintances] used Suboxone, but none of them were prescribed it. It was just from the street. One of the biggest fallacies of it is people will buy, like I said, two strips of Suboxone, and they’ll be like, “I’m gonna wean myself off and I obviously can’t do it with heroin, so I’m gonna do it with Suboxone.” And they’ll start at two milligrams for two days and bring it down to 1.5 or whatever. And most of them get a week into it before they’re back on the street. That’s when I’ve seen the most of it is people like, “Yeah, I’m gonna get Subs because I’m gonna wean myself and get clean.” It never works (MKE203)

Even when participants were not discharged from MOUD treatment, the threat of punishment was often enough for participants to discontinue treatment when they had used other drugs and were afraid it would be detected in urine drug screens.

Participant: I went to the [residential] treatment center. That’s where I was clean for about eight months. While I was in the program, they put me on Suboxone. So, when I got out of the program, they placed me in like sober living… And I had to go once a week, have a little group and they would prescribe me the weekly prescription and then that was that. And I’d take it home, take it once a day and, for the most part, it helped a lot.

Interviewer: Did you have a reason for why you stopped with suboxone?

Participant: No. I just started messing up doing other things. It was completely helpful for the opioids, but I started drinking and using cocaine. And one time I got a dirty urine when I went to one of my groups. The doctor told me if I get another one, he’s gonna have to put me back into like a 30-day program. So, I knew it was dirty the next time I was supposed to go, and I just stopped going and then that’s what led to me being back here. (HAR201)

While the doctor could not commit the participant in residential treatment without his consent, the doctor implied that he would no longer treat him with buprenorphine if he did not comply. Residential treatment allows very little time for patients “on the outside” and disrupts employment, education, and family responsibilities.

Many of these regulations were in place both for office-based buprenorphine and clinic-based buprenorphine and methadone. The advantage of buprenorphine is the ability to get prescriptions that can be taken at home without traveling every day to a clinic for their dose like with methadone. However, many participants reported that some buprenorphine providers were as strict as methadone clinics and required frequent visits and urine drug screens.

Participant: Well, I’d have to say that the [Name of Program] is crap…. He would – dosing them for suboxone, so instead of just giving you a prescription for suboxone, he’s treating it like methadone and making you come in everyday to get dosed so that he can get more drug tests out of you and everything like that. So, places like [that] … where the pee test and everything like that so that they could make more money…. And they told me there that pee is like gold, so all these places make money easily off of our urine. I know it’s disgusting, but – yes, urine I said. I didn’t stay probably long enough for that. (WAU210).

One participant reported that his insurance would not pay for the urine drug screens and so he discontinued buprenorphine and began buying them on the street. (WAU211).

While some participants resented the frequent monitoring, like the participant above, others appreciated it and felt that it helped to keep them accountable. Some participants felt that low-barrier approaches, where the doctor wrote a prescription was irresponsible and showed a lack of care for the patient.

Participant: Getting yourself to like the medications and eventually figuring out about this place… This is probably the one reason why I continue to stay clean.

Interviewer: What do you mean? Can you explain that a little bit more?

Participant: Because they keep me accountable just by coming and making sure that I’m drug tested. I mean, because you can’t fail. I mean, you can’t really pass a drug test if you’re dirty. So, just being accountable for my actions and – the suboxone and everything. Like having – the suboxone helps because a lot of places if you want to be put on suboxone, you have to pay out of pocket $200.00 to $300.00. Then, yeah, we’ll give you a month’s worth. They don’t care if you’re clean or dirty necessarily. They don’t care if you’re using; they don’t care if you sell them as long as you get back to them. I didn’t have that money. They didn’t take my insurance. This place did. And you know, they only give you x amount. And you have to reach up to, you know, getting you know once a week or once a month. So, and that’s what I liked a lot too. You know you have to be accountable for your actions these days. (WAU209)

Other participants reported being kicked off of MOUD because they missed an appointment or because they failed to show the appropriate number of pills during a random pill count.

Participant: Well, I was in the clinic, Suboxone clinic, for a little while and then I got kicked out because I let a friend come to my house and I thought he was my friend, and he stole my medicine. And the clinic somehow found out, called me in for a pill count. So, they kicked me out and I had to start buying it for, like, four months before I got into another clinic and that’s how that went on. (HAZ214)

Participant: Yeah, I was on the Suboxone, actually for a little while. And I don't know, it actually was fairly successful with it, but the problem was I would pick up my prescription, maybe a months’ worth at a time. And I would go to the doctor once every few months, and it was, this is going pretty good. And this one day, my dog – I had an appointment to see my doctor, and then, my dog took off, as I was walking out the door to go to my appointment. And I live out in the country. She’s deep into the woods. And instead of leaving her out in the woods by herself, I stayed there and got her back in the house. So, a half hour later, actually I called. He was mad, so I told him, hey, I have an appointment and I’m missing it. And he said, you can’t get an appointment for another two months or something like that. And I was kicked out of the program because I missed an appointment. But the dog took off. So, yeah. I took all the Suboxone that I had so I could wean myself off of it, which it worked. [MAR215]

Some participants reported that they were encouraged or required to attend group sessions, like NA or AA while on MOUD. While some participants thought it helped them develop tools to avoid triggers and deal with cravings, others thought that the groups themselves triggered cravings.

Participant: When I was serious about going clean, that one time I told you about, you know they pushed the NA meetings on you and the 12-step program and all of that and tried the meetings and, to tell you the truth, going to the meetings just made you talk about drugs and got it on your mind and made you think about it. You know, like hearing everybody’s stories and it just put it in your mind. When I was trying to go clean, I tried to stay busy and I just tried to stay away from certain people and places and that worked for me for a while, you know? It worked better than going to the meetings I felt because, like I said, when you go to the meetings, people are talking about it. It gets in your head…. If you stay busy, you got other things on your mind. You got work, you got going on dates or whatever, dating and, you know, you got other things on your mind other than drugs (MKE217)

Some participants reported avoiding drug treatment programs because they required group attendance, like the woman below.

Interviewer: So, what do you think about drug treatment programs?

Participant: I really don’t like them.

Interviewer: Why?

Participant: Why should I get to a drug treatment program? Everybody you know is on drugs? I gotta hear your story, you gotta hear my story. What about you say some shit that I ain’t never done before? I’m gonna go try that. I don’t wanna hear that. I’m here to get some help. I don’t wanna hear nobody’s stories. I don’t wanna tell no stories. I just wanna know how to get off and stay off the shit (MKE 209)

The constant surveillance and strict adherence to seemingly arbitrary rules added to participants' feelings of stigma and as people who could not be trusted.

Interviewee: I go to a suboxone clinic, and I know they've been pretty good. The doctors are really nice, and the facility administrators are really nice. But I've found that the place that I go to at least, the staff, they treat you like you're just a dirty junkie. They literally treat you like shit. Like right now I'm going to my clinic because I was late for my appointment the other day and they won't reschedule me an appointment. I have to go up here and sit until the doctor has a no-show, and then if he has a no-show, then they see me. And until then, I don't get my medicine. And this is being 15 minutes late, not like missing the entire appointment. (LEX208)

Another participant reported being discharged for allegedly selling his methadone, with no evidence other than another client’s report.

## Discussion

Results from this study highlight a need for more compassionate, harm-reduction oriented services. Participants’ experiences with drug treatment offer insight into the low retention rates for medications to treat opioid use disorder observed in numerous studies. Many studies have examined individual level factors that are associated with poor retention in MOUD and other drug treatment programs including polysubstance use, more severe opioid use disorder and homelessness [18, 29, 31, 32, 52]. The implication from these studies seems to be that polysubstance users are unable to adhere to MOUD and that they require additional services, including treatment options, such as contingency management, to address their other substance use and psychosocial needs. However, many of these barriers may very well be at the organizational level with polydrug users more likely to fail urine drug screens and be discontinued in treatment [31]. Many times, these additional drugs did not decrease the safety of MOUD, and it was not clear that programs were offering services to address other substance use problems. However, even when the polydrug use is contraindicated with methadone or buprenorphine as with, for example, benzodiazepines, it is not clear why failing to achieve complete abstinence from drugs is reason for discharge. If substance use disorders are considered chronic diseases in which relapse is expected, then discontinuing treatment for failing to achieve abstinence makes little sense. When MOUD is discontinued, some participants reported going back to illicit drugs, others bought MOUD on the street to try to continue treatment or used MOUD to withdraw from opioids.

Results also suggest that there may be a mismatch between providers’ and clients’ treatment goals. Abstinence may not be clients’ goal as some may want to decrease their tolerance or to avoid going through withdrawal every morning [53]. For these clients, getting up every day and living a “normal” life without having to constantly hustle for money to buy drugs was a significant improvement in their lives. Other goals, such as employment or re-establishing relationships with family, may be more important. Many qualitative studies have explored differences between patient and clinic treatment goals and have suggested a more flexible approach to drug treatment which takes patients’ personal goals into account [54]. These goals could be part of a treatment plan collaboratively developed with patients and providers. Many social work interventions, for example, permanent supportive housing, use a similar approach [55].

Many participants sought drug treatment from detox centers. In some cases, detox was the entry point for continued outpatient or MOUD treatment; other times it was not followed with any treatment. Some researchers have suggested adapting detox to initiate MOUD and link patients to community MOUD providers [35]. However, medically assisted inpatient detox is very expensive compared to outpatient MOUD induction [49]. Further, most detox centers fail to link patients to follow-up treatment [39–41]. Research has shown that detox without follow-up treatment increases risk of overdose [38, 56]. It is not clear why the practice of detox for OUD persists, although some suggest that inpatient detox can be useful for people experiencing homelessness [57]. In addition, detox is often covered by insurance. In our study, detox was covered by Medicaid in Connecticut and Kentucky. Wisconsin’s Medicaid did not cover detox for OUD but was often paid for by County block grants [57]. De-implementation interventions aim to eliminate or reduce the use of ineffective or harmful treatments with priority given to medical practices that are harmful and those that affect more people [58, 59]. Detoxification meets these criteria in abundance, but to our knowledge, no systematic, multi-level interventions have been developed to de-implement the practice. As opioid-related overdoses continue to rise, interventions to de-implement detoxification and replace them with appropriate treatments are urgently needed. De-implementation interventions are in their infancy, but preliminary studies suggest that interventions need to target multiple levels: policy, insurance, healthcare system, clinician, characteristics of the practice to be eliminated or replaced and patient factors [60, 61].

Part of the persistence of detox likely stems from MOUD stigma. People with OUD who hold stigmatizing attitudes may want to continue in abstinence based non-MOUD treatment, or to quit on their own. MOUD stigma is held by drug treatment professionals, people with OUD and the larger community. Among providers this is often manifested as a reluctance to prescribe or recommend MOUD, and among  people with OUD, it manifests as a reluctance to take MOUD and a preference for “abstinence only” models [16]. People with OUD and their family and friends often feel that MOUD is “just another drug” and that those who use it are not in recovery [20]. Behavioral interventions are often used successfully with MOUD, but many patients and drug treatment providers promote drug abstinence [16].

Interventions are needed to address MOUD stigma. While some interventions have begun to address MOUD stigma among providers who may prescribe MOUD and pharmacists who dispense, to our knowledge, no intervention has addressed MOUD stigma among people with OUD. Addressing MOUD stigma among people with OUD may increase demand and, thus, contribute to bridge the current MOUD treatment gap. Social network interventions have been shown to be effective in changing behavior and attitudes of hidden and marginalized communities and could be used to address MOUD stigma. Of particular relevance to the proposed intervention, social network interventions with sexual and gender minority individuals have been shown to reduce HIV risk behaviors and increase PrEP uptake and maintenance [62, 63]. Among people who inject drugs, social network interventions have been successful at reducing injection risk behaviors and increasing use of syringe service programs (SSPs) [64, 65].

Results of this study also show that the implementation of buprenorphine treatment is often just as strictly monitored as methadone. Participants reported programs that required them to come into the clinic or doctor’s office multiple times a week and to take frequent urine drug screens. This eliminates some of the advantages of buprenorphine over methadone. Historical regulations of methadone clinics are probably also unnecessarily harsh, as the switch to home delivery of multiple doses of methadone during COVID-19 when many MOUD clinics were closed demonstrated [27, 28]. There is little reason for these regulations with buprenorphine which is much safer than methadone and our results suggest that such requirements are another barrier to treatment retention. While some participants reported that they appreciated frequent drug screens because they held them accountable, more patient-centered approaches to drug treatment could allow for more frequent screening for those who find it helpful while not requiring it for those who do not. Further, such practices can contribute to stigmatization of people who use drugs as they are seen as not trustworthy.

## Conclusions

In this unprecedented epidemic of OUD and opioid overdoses, there is an urgent need to examine and improve current practices to improve treatment initiation and retention. Many participants blamed themselves for treatment failure, but it appears there is much room for improvement. Medicine in general is moving away from autocratic decision-making by providers to shared decision making. People who use drugs can and should be trusted to help decide on their treatment and the outcomes they desire.

## Data Availability

Data from qualitative interviews are not publicly available to the public due to concerns to protect confidentiality in in-depth interviews but are available from the corresponding author in aggregate on reasonable request.
